# Reciprocal activation of gastrocnemius and soleus motor units is associated with fascicle length change during knee flexion

**DOI:** 10.14814/phy2.12044

**Published:** 2014-06-11

**Authors:** Benedikt Lauber, Glen A. Lichtwark, Andrew G. Cresswell

**Affiliations:** 1 Department of Sport and Sport Science, University of Freiburg, Freiburg, Germany; 2 School of Human Movement Studies, The University of Queensland, Brisbane, Queensland, Australia

**Keywords:** MU recruitment, muscle‐tendon unit length, triceps surae

## Abstract

While medial gastrocnemius (MG) and soleus (SOL) are considered synergists, they are anatomically exclusive in that SOL crosses only the ankle, while MG crosses both the knee and ankle. Due to the force‐length properties of both active and passive structures, activation of SOL and MG must be constantly regulated to provide the required joint torques for any planned movement. As such, the aim of this study was to investigate the neural regulation of MG and SOL when independently changing their length by changing only the knee joint angle, thus exclusively altering the length of MG fibers. MG and SOL motor units (MU) were recorded intramuscularly along with ultrasound imaging of MG and SOL fascicle lengths, while moving the knee through 60° of rotation and maintaining a low level of voluntary plantar flexor torque. The results showed a reciprocal activation of MG and SOL as the knee was moved into flexion and extension. A clear reduction in MG MU firing rates occurred as the knee was flexed (MG fascicles shortening), with de‐recruitment of most MG MU occurring at close to full knee flexion. A concomitant increase in SOL MU activity was observed while no change in the length of its fascicles was found. The opposite effects were found when the knee was moved into extension. A strong correlation (ICC = 0.78) was found between the fascicle length at which MG MUs were de‐recruited and subsequently re‐recruited. This was stronger than the relationship of de‐recruitment and re‐recruitment with knee angle (ICC = 0.52), indicating that in this instance, muscle fascicle length rather than joint angle is more influential in regulating MG recruitment. Such a reciprocal arrangement like the one presented here for SOL and MG is essential for human voluntary movements such as walking or cycling.

## Abstract (precis)

This study show that under dynamic conditions, drive to the medial gastrocnemius muscle is modulated such that motor unit de‐recruitment occurs with progressive medial gastrocnemius fascicle shortening and recruitment occurs with progressive lengthening. Furthermore, the results also indicate that muscle length rather than joint angle accounts for the changes in medial gastrocnemius recruitment during knee flexion and extension. This has important consequences for our understanding of the control of multiarticular joint muscles.

## Introduction

During everyday movements like walking and cycling, lower limb muscles undergo continual changes in overall length as joints change their position. Due to the well‐known force‐length properties of both active and passive structures within a muscle, neural activation must therefore be constantly regulated to provide the required joint torques needed for these movements and other similar movement tasks (Kaya et al. 2003; Donelan et al. 2009). The medial gastrocnemius (MG), lateral gastrocnemius (LG), and soleus (SOL) muscles are of special interest in this context because unlike SOL, which only crosses the ankle joint, MG and LG cross both the knee and ankle joints and their muscle‐tendon unit lengths and consequently force‐producing capacity are dictated by the position of both joints. Even though these muscles act as agonists, their neural activation has been shown to change depending on joint position. For example, a study by Cresswell et al. (1995) observed a graded reduction in neural drive to MG as it was incrementally shortened by changes in knee joint angle, despite subjects' being asked to produce a maximal voluntary plantar flexor effort. Soleus electromyography activity remained the same for all knee angles and it was assumed that there was no change in its MTU and fascicle lengths.

A similar reduction in MG neural drive at short muscle lengths was reported for submaximal isometric plantar flexions at different knee joint angles (Kennedy and Cresswell 2001). In that study, subjects were asked to perform isometric plantar flexion ramp contractions at short and long gastrocnemius MTU lengths, while single MU's were discriminated in MG. It was shown that when MG MTU length was short (i.e., during knee flexion), a higher level of neural drive, as measured by MU recruitment threshold, was required to recruit MG MU's. Possible mechanisms underpinning the change in MG recruitment threshold were postulated, with (1) higher threshold or faster MU's being selectively activated at shorter muscle lengths, while lower threshold MU's were inhibited; or (2) there was an overall change in recruitment threshold of the MG pool, with higher drive required to activate the same population of units. A technical limitation of the study that prevented the authors from determining which of the above mechanism was relevant, was the inability to confirm that the same MU were recorded at the different muscle lengths. This was due to the fact that stiff concentric needle electrodes were used to isolate unit firings, and to avoid damaging the muscle when changing muscle length the needles were removed and reinserted. To overcome that limitation it would be necessary to follow the firing of the same MUs while the length of MG is being continuously altered and plantar flexion torque is kept constant. Furthermore, reports from other studies also show changes in motor unit activity in relation to changes in fascicle length depending on the knee angle (Altenburg et al., 2009), ankle angle (Hwang & Cho, 2004; Pasquet et al., 2005), and different levels of surface EMG activity when changing the ankle or knee joint angle (Arampatzis et al. 2006).

Another confounding issue relates to the fact that while knee joint angle is a reasonable measure for MTU length, it can be a poor correlate of muscle fascicle length. Recent studies have shown that passive compliant structures, such as the Achilles tendon, can act to decouple the relationship between joint angle (MTU length) and muscle fascicle length (Arampatzis et al. 2006; Maas and Lichtwark 2009; Hoffman et al. 2012). This decoupling is particularly heightened when increased activity in the muscle increases results in even greater stiffness of the active muscle fibers. Additionally, tendon length and its associated passive stiffness can also change as the MTU changes length during changes in joint angle and changes in muscle activation. Recent advances in ultrasonography and image analysis now provide a method to directly measure changes in muscle fascicle length, which are indicative of changes in muscle fiber length (Cronin and Lichtwark 2013). In this study, we utilize this technique along with intramuscular electromyography to determine whether MTU or fascicle length is the determining factor for the previously observed recruitment and de‐recruitment of MG MUs as the knee is moved into flexion.

Therefore, the aim of this study was to identify whether the MG and SOL muscles would be activated in a reciprocal manner when rotating only the knee joint and while maintaining a constant level of voluntary plantar flexor torque. If MG activity was found to be modulated with changing knee joint angle, a secondary aim was to determine whether such modulation is related to MTU length (i.e., joint angle) or muscle fascicle length. Given that MG has been shown to operate primarily on the ascending limb of the length–tension relationship (Hoffman et al. 2012), our a priori supposition was that de‐recruitment of MG MUs would occur when MG fascicles reached unfavorable lengths for force production, that is shorter lengths during knee flexion and re‐recruit during knee flexion.

## Methods

### Subjects

Ten healthy male subjects (27.7 ± 5.3 years; 179.8 ±4.9 cm, 79.3 ± 10.5 kg, mean ± standard error of the mean (SEM) with no history of neurological injury or disease agreed to participate in this study. The procedures were approved by the local university ethics committee and performed in accordance with the Declaration of Helsinki. All subjects gave written informed consent prior to participating.

### Set‐up

**Figure 1.**
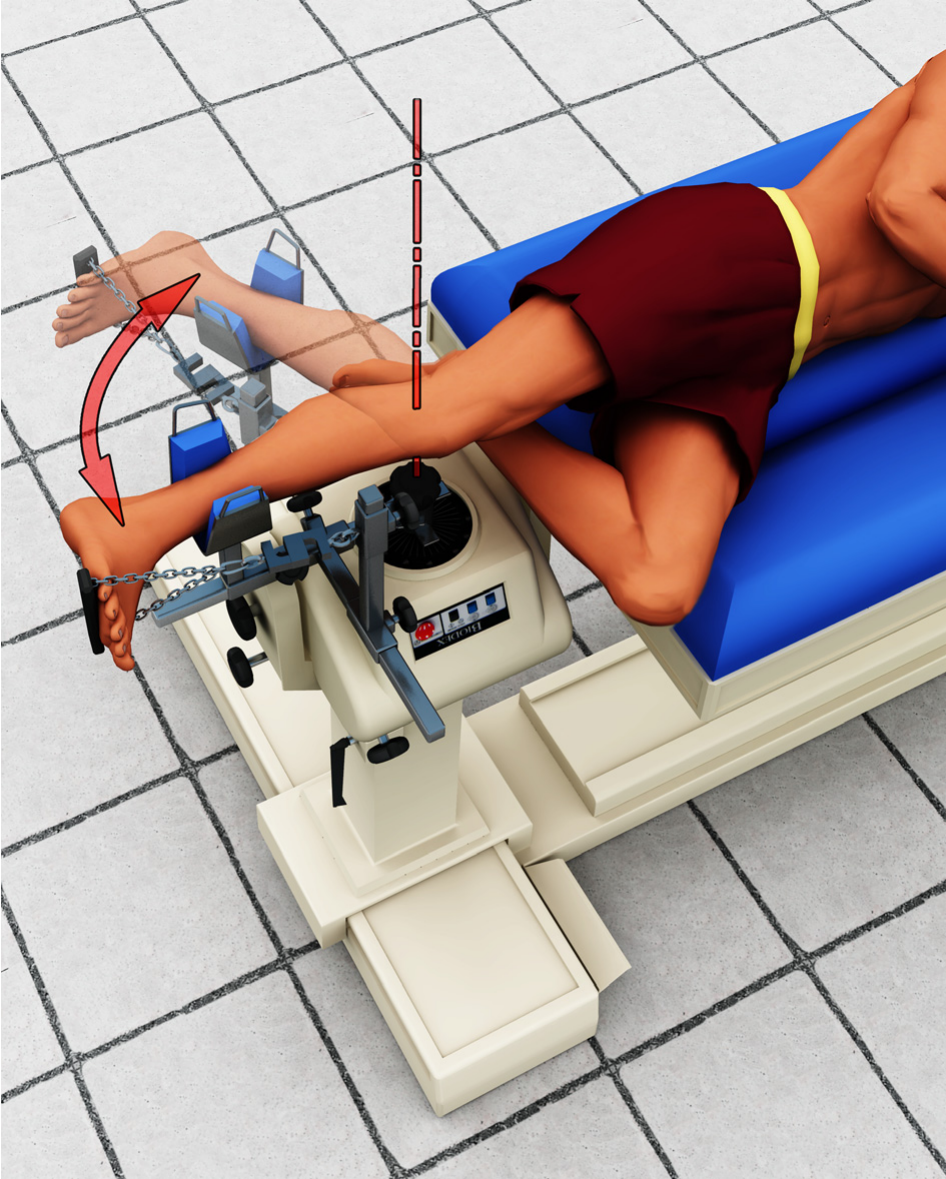
Illustration of the experimental setup. Subjects were lying on their side with their ankle fixed at approximately 90^°^ while their knee was passively rotated over 60^°^ into flexion and back to extension. This position enabled ultrasound imaging of the medial gastrocnemius and soleus muscles. Single MUs were recorded from medial gastrocnemius and soleus using fine‐wire electrodes. Visual feedback of ankle torque was provided to the subject so that they could maintain a required plantar flexion torque of approximately 5% of a maximal voluntary contraction.

The experimental setup has been described previously (Tokuno et al. 2012). In brief, subjects were positioned side‐lying (Fig. 1) with their right hip extended and their right knee aligned with the axis of a torque motor (Biodex System 3, Biodex Medical Systems, New York). The right shank lay on a padded support that was attached to the lever arm of the torque motor. The right ankle was positioned and held between 5° and 10° of dorsiflexion using linked chain attached to a small plate beneath the ball of the foot. Successful positioning of the subject prevented any change of ankle angle as the knee was rotated through a specified range of motion by the torque motor. Subjects remained in this position throughout the entire experiment which took approximately 1.5 h.

### Protocol

The subject's torso and thighs remained motionless while their knee was passively rotated through 60° into flexion and extension by the torque motor at a constant angular speed of 4°s^−1^ starting from an almost fully extended knee position (0°). Five complete cycles were performed with each cycle having a 2‐s pause at each end point. At the same time, and throughout the entire five cycles, subjects were required to produce a constant plantar flexion torque (≈2.5% of a maximum voluntary contraction; ≈4 Nm on average). Real‐time plantar flexion torque output was displayed on a computer screen placed in front of the subjects to provide visual feedback in order to maintain the target force. These trials were performed while single MU activity was simultaneously recorded using fine‐wire intramuscular electromyography (EMG) from the MG and SOL muscles (see below). After five cycles, the position of the electrodes was changed by gently pulling on the wires so they moved more superficially within the same muscles and five additional cycles were performed. This was repeated approximately three to four times until the electrodes were no longer in the muscle tissue. After these trials, the fine‐wire electrodes were removed and five additional cycles were performed in an identical fashion while ultrasound measurements of MG and SOL fascicles were made at the location where the electrodes were implanted.

### Electromyographic recordings

Fine‐wire intramuscular electrodes were inserted at locations according to the Seniam^®^ (Enschede, The Netherlands) guidelines in the most bulging part the muscle belly of MG and for SOL, at 2/3 of the line between the medial condyle of the femur and the medial malleolus. Electrodes were fabricated using Teflon‐coated wires made of stainless steel, 50 *μ*m diameter (California FineWire) with a detection region of 1 mm. One delivery needle (23‐gauge, 32 mm) containing two wires with their detection areas approximately 3–4 mm apart was inserted into each muscle under sterile conditions. The delivery needles were removed leaving the pairs of wires remaining in the muscle. The position of the electrodes was then adjusted by lightly pulling at the wires to obtain an improved signal‐to‐noise ratio during weak, brief contractions. A single Ag–AgCl surface electrode (diameter 10 mm, Tycell Healthcare Group LP, Hampshire, UK) was placed over the lateral condyle of the tibia and used as a reference electrode. All EMG signals were amplified 1000 times and band pass filtered between 50 Hz and 4 kHz (NL844 and NL105, respectively, Digitimer Ltd, Hertfordshire, UK) before analogue to digital conversion at 20 kHz (Spike2 and Power 1401, Cambridge Electronic Design, Cambridge, UK).

### Ultrasound measurements

Ultrasound B‐mode images were recorded for MG and SOL muscle fascicles while the knee was rotated by the torque motor. A PC‐based ultrasound system (Echoblaster 128, UAB ‘Telemed’, Vilnius, Lithuania) using a flat‐shaped ultrasound probe (96‐element, linear probe, frequency of 7 MHz, field of view of 60 mm, capture frequency of 80 Hz) was used to image the fascicle length of MG and SOL. The probe was strapped to the shank using elastic bandage to prevent any unwanted movement that would invalidate the measurements. MG was imaged in the mid‐belly region, while the SOL was imaged distal to the gastrocnemius muscle–tendon junction, slightly lateral to the midline. For both muscles, the ultrasound transducer was aligned such that the plane of the image was perpendicular to the deep fascia so that clear continuous muscle fascicle lines could be imaged (Hoffman et al. 2012). A logic pulse was used to synchronize the ultrasound recordings with the start of the motion, the torque measurements and the EMG recordings.

### Torque and knee position measurements

Plantar flexion force was measured using a S‐type load cell (Model STC, Scale Components, Slacks Creek, Australia) placed in series with the cable attached to the foot and aligned parallel to the long axis of the tibia. The force signal was DC amplified and low‐pass filtered at 5 Hz and analogue‐to‐digitally converted at 1 kHz (Spike2 and Power 1401, Cambridge Electronic Design, UK). Plantar flexion torque was calculated from the force signal and the known perpendicular distance from the load cell to the estimated center of the axis of rotation of the ankle joint. Angular position of the dynamometer was processed using the same settings as for the force measurement and was used to describe the angular position of the knee.

### Data analysis

#### Motor unit discrimination

Discrimination of single MU potentials was performed offline using the template‐matching features of Spike2 software (Cambridge Electronic Design, UK). As MUs can be assumed to discharge fairly regularly, such that the next instant of a MU discharge can be predicted by using the mean interspike interval (ISI) of previous discharges (Oya et al. 2009), wrongly classified MUs were visually identified as gaps or unusually high‐frequency firings in the instantaneous firing rate record and subsequently manually corrected finding the template that best fitted the unit or creating a new unit template. Recruitment threshold for each MU was determined by iterating a 0.5‐s window forward in 1‐ms intervals until the coefficient of variation (CV) of the ISI separating the potentials within the window was <50%. At this point the time of the first potential in the moving window was used for threshold determination. De‐recruitment threshold was determined in the same manner but by moving the window backwards from the last segment of the signal in 1‐ms steps (Moritz et al. 2005; Oya et al. 2009; Kelly et al. 2013). The mean firing rate at recruitment and de‐recruitment was calculated over the same 0.5‐s window that included the first potential defining the recruitment or de‐recruitment threshold. Knee angle and MG fascicle length corresponding to the times of recruitment and de‐recruitment thresholds were then calculated. Mean MG firing frequency at recruitment and de‐recruitment was calculated over the initial and final five spikes of the flexion and extension phases. SOL activity was measured as the sum of all MU spikes discriminated in 1‐s bins across a complete flexion and extension cycle.

#### Muscle fascicle length measurement

Image processing was performed offline using custom written Matlab scripts (Mathworks Inc., Chatswood, MA). This method included the use of an automatic tracking algorithm (Cronin et al. 2011) which has been shown to have high repeatability during both active and passive conditions (Gillett et al. 2013) and was used in the same way as described by Tokuno et al. (2012). Muscle fascicle length was defined as the straight‐line distance between the deep and superficial muscle aponeurosis within the middle of image where the full length of the fascicle could be visualized. Movement of the end points of this line were calculated from one frame to the next, which accounts for any change in pennation angle when estimating fascicle length from contractile tissue displacement. To be able to compare between participants with different absolute fascicle lengths, fascicle length was normalized to the shortest fascicle length during the flexion–extension cycle.

### Statistical analyses

For each subject, individual recruitment and de‐recruitment thresholds were calculated for the flexion and extension phases. A Student's paired t‐test was computed to compare averaged data across subjects for fascicle length knee angle at de‐recruitment and recruitment. Because we could not follow all units across all five cycles, we determined thresholds for each unit for each flexion and extension phase and included all values into the correlation analyses. Knee angle and fascicle length at recruitment and de‐recruitment threshold were similarly determined. To test whether the de‐recruitment and recruitment of MG MUs were related to muscle fascicle length or knee joint angle, we calculated the intraclass correlation coefficient (ICC) of fascicle length and knee angle at de‐recruitment and recruitment. SOL EMG activation (measured as the sum of spikes over 1‐s bins) was averaged across the five cycles and subjects and then normalized to the bin with the highest number of spikes (mean ± standard error of the mean (SEM)). Student's paired t‐tests were computed to compare averaged data across subjects for MG EMG firing frequency and number of spikes measured in SOL during the flexion–extension cycle. For all analysis, the level of significance was set at *P ≤ *0.05.

## Results

A total of 88 MG MUs were successfully discriminated from all flexion–extension cycles, which produced 264 measureable de‐recruitment recruitment thresholds. While some single MUs could be visually identified from the SOL recordings, the purpose of this recording was to evaluate general modulations in drive to the SOL motoneurone pool, and as such individual MUs were not template matched or followed in SOL.

**Figure 2.**
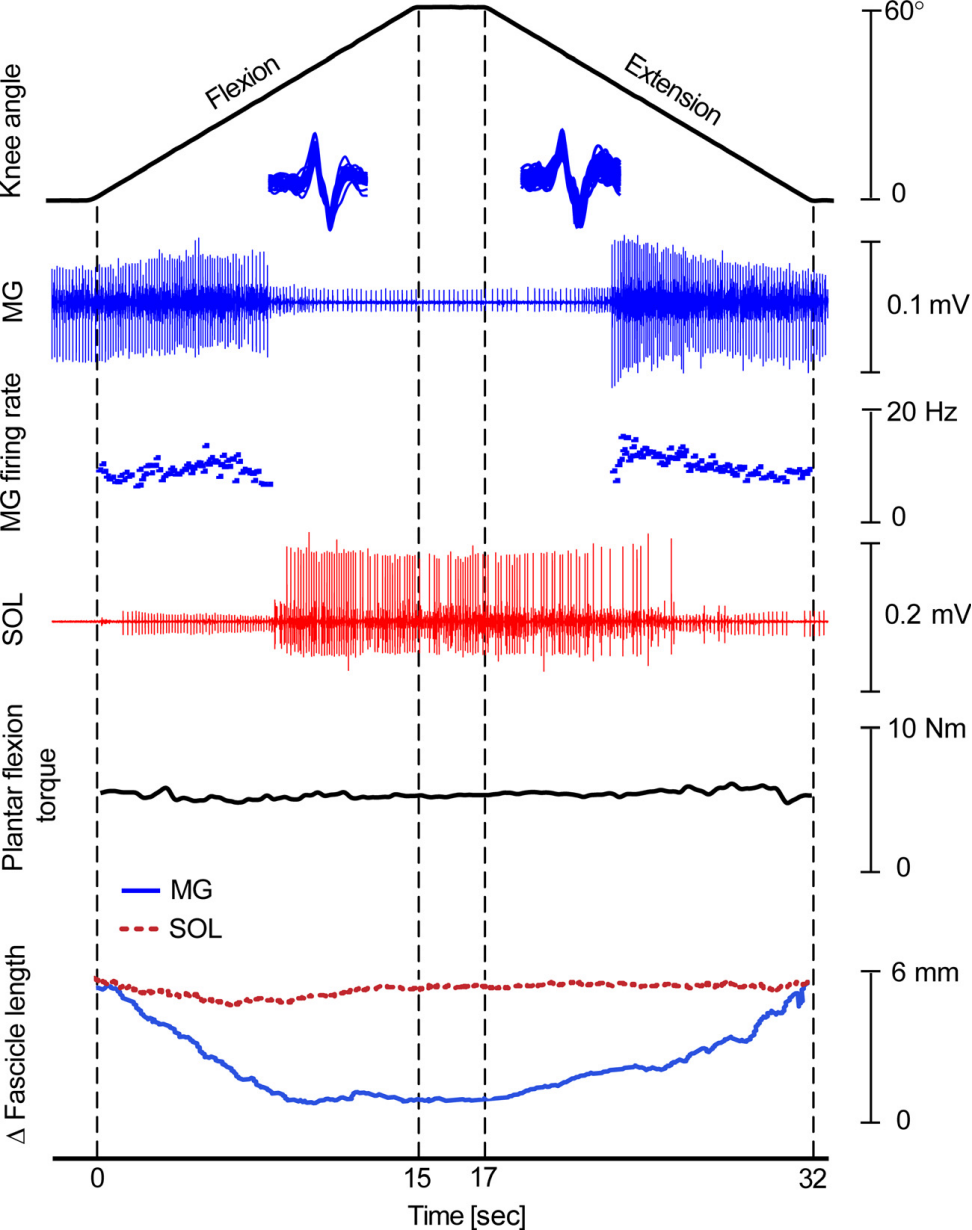
Example data taken from a single subject. From top to bottom, knee angle, medial gastrocnemius (MG) activation, MG MU firing frequency for a single identified MU, soleus (SOL) activation, plantar flexion torque, MG and SOL fascicle length changes are shown. During knee flexion (0–15 s), a MG MU was de‐recruited while SOL EMG activity increased. Knee flexion resulted in a shortening of MG fascicles (solid blue) while SOL fascicle (dotted red) remained at a relatively constant length. The opposite pattern was observed during knee extension (17–32 s).

Representative data from a single subject are shown in Fig. 2 and demonstrate the effect of passively rotating the knee while maintaining a constant low level of ankle plantar flexor torque on MG MU de‐recruitment and recruitment. The instantaneous firing rate of an isolated MG unit is also shown, as well as an intramuscular EMG recording from SOL, MG, and SOL fascicle lengths and plantar flexor torque. The example shows the typical finding of MG units being de‐recruited as the knee progresses into flexion and MG fascicle length shortens. Recruitment of the same unit occurs as the knee moves into extension and MG fascicles are lengthened. Analysis of MG instantaneous firing rates revealed an initial increase in firing rate as the knee flexed and MG began to shorten, followed by a brief decrease just prior to de‐recruitment. Similarly, on recruitment, MG firing rates briefly increased to a maximum and then decreased through the remaining part of the lengthening cycle. SOL activity was reciprocal to that of MG, with increasing firing rates and/or recruitment of additional units during knee passive flexion and de‐recruitment and/or decreasing firing rates during passive knee extension. These changes occurred in SOL despite relatively little change in SOL fascicle length or voluntary plantar flexor torque.

### Muscle fascicle length and torque output

**Figure 3.**
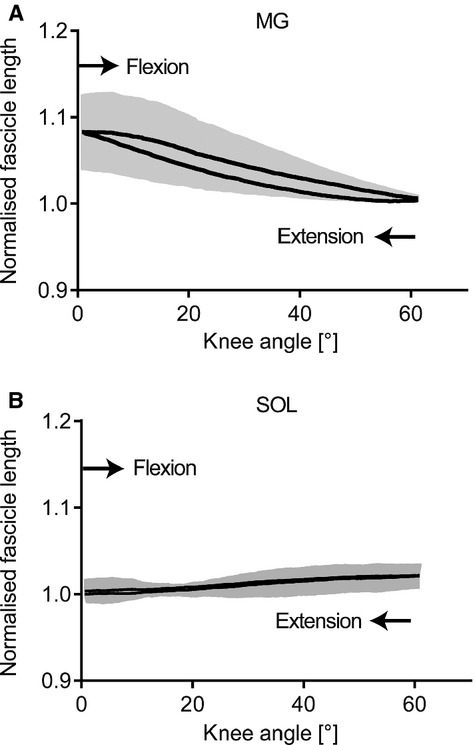
Group mean average of the normalized fascicle length. Data for fascicle length are plotted against knee angle for medial gastrocnemius (MG, A) and soleus (SOL, B). Gray‐shaded area represents ± standard error of the mean about the mean. During the course of the knee flexion, MG fascicle length decreased followed by an increase during knee extension. SOL fascicle length remained relatively unchanged throughout the flexion–extension cycle.

Grouped data revealed that MG muscle fascicle length decreased by 3.5 ± 1.9 mm (*P *≤**0.05) during the course of knee flexion. MG fascicles increased to their starting length during knee extension; however, hysteresis was evident in the grouped data with slightly longer MG fascicle lengths occurring during flexion than extension for the same knee angle (Fig. 3A showing normalized data). Negligible length change was seen in SOL fascicles with grouped data resulting in a length change of 1.0 ± 0.2 mm over the complete cycle (Fig. 3B showing normalized data). Subjects were able to maintain a constant submaximal plantar flexion torque across both movement phases, with no statistical difference (*P *=**0.6) in plantar flexion torque between the flexion and extension phases (3.8 ± 0.6 Nm and 3.9 ± 0.8 Nm, respectively).

### MU recruitment and de‐recruitment

**Figure 4.**
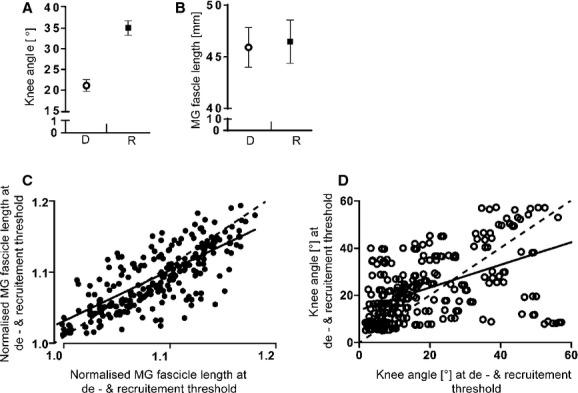
De‐recruitment versus recruitment threshold for fascicle length and knee angle. De‐recruitment (○) versus recruitment (■) threshold was significantly different (*P *≤**0.05) for knee angle (A) but not medial gastrocnemius (MG) fascicle length (B) (*P *>**0.3). The intraclass correlations (ICC) of the 264 recruitment versus de‐recruitment thresholds were stronger for fascicle length (C, ICC = 0.78) than knee angle (D, ICC = 0.52).

Results from the grouped data showed MG MUs being de‐recruited during knee flexion at a knee angle of 21.1 ± 5.4°, which was significantly different from the knee angle when the same unit was re‐recruited (35.1 ± 4.3°, *P *≤**0.05, Fig. 4A). When de‐recruitment and recruitment were evaluated against MG fascicle length rather than knee angle, de‐recruitment and recruitment occurred at mean lengths of 45.9 ± 1.9 mm and 46.5 ± 2.1 mm, respectively, which were not statistically different from each other (*P *>**0.3, Fig. 4B). ICC values for recruitment versus de‐recruitment threshold for fascicle or knee angle revealed a stronger relationship for fascicle length (ICC = 0.78, Fig. 4C) than knee angle (ICC = 0.52, Fig. 4D). The mean MG firing rate at de‐recruitment was found to be significantly higher than its firing rate when re‐recruited at the same fascicle length, with values of 6.3 ± 2.7 Hz and 4.4 ± 1.8 Hz (*P *≤**0.05), respectively.

**Figure 5.**
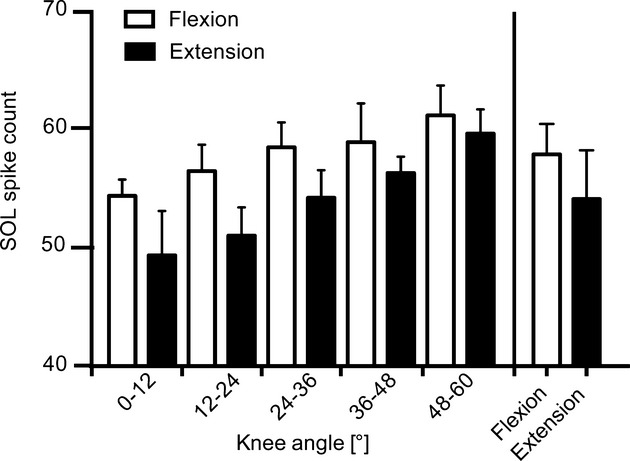
Group mean average of the soleus (SOL) activation: SOL activation was measured over 12° intervals by counting the total number of MU spikes within each bin for flexion (open bars) and extension (filled bars). Although not significant, the number of SOL spike counts was greater for flexion than extension for each interval. This was also the case for the mean count across each of the phases (bars furthest to the right).

Grouped data revealed a graded modulation of SOL drive, with increasing SOL EMG activity with increasing knee flexion and decreasing activity during knee extension. The change in SOL EMG occurred despite no change in SOL fascicle length or plantar flexion torque throughout the complete cycle. Mean SOL activity was overall lower for knee extension than for knee flexion (Fig. 5, bars furthest to the right).

## Discussion

The aim of this study was to identify whether MG and SOL muscles would be activated in a reciprocal manner when rotating the knee joint and while maintaining a constant level of voluntary plantar flexor torque. A reciprocal relationship between MG and SOL was identified as the length change of these muscles was decoupled from each other. As MG fascicles shortened with knee flexion, an increase in SOL activity occurred to compensate for a loss of force production from a shorter MG. De‐recruitment of the initially recruited low threshold MG motoneurones seemed to be driven by a reduction in MG muscle fascicle length rather than knee position. Although not revealed here, the current findings confirm the existence of a neural mechanism whereby MG fascicle lengths are used to provide the central nervous system with the physiological state of the muscle and as such regulate the balance of inhibitory and excitatory inputs to its motoneurone pool.

This study builds upon the earlier finding of significantly increased levels of SOL EMG activity and plantar flexor torque that corresponded to the onset of firing of MG MUs during voluntary ramp contractions when the knee was flexed to 90^°^ (Kennedy and Cresswell 2001). While no mechanism was given to support that finding, the changes were credited to increased inhibition of the MG motoneurone pool driven by the diminished force‐producing capability of the MG muscle at short lengths. Peripheral afferent inputs were indicated as likely contributors to the effect, with reduced Ia‐afferent input via increased presynaptic inhibition through increased activation of group II, III, and IV afferents, as well as effects from cutaneous and joint afferents being suggested.

The findings of this study are additionally supported by at least two earlier studies showing a more than 50% decrease in MG surface EMG as the knee is incrementally moved from positions of full extension to flexed positions of 60^°^ or more (Cresswell et al. 1995; Tamaki et al. 1997). In both studies, SOL surface EMG either increased significantly for submaximal efforts (Tamaki et al. 1997) or remained the same for maximal efforts (Cresswell et al. 1995). Interestingly, less of an effect was observed in the lateral gastrocnemius muscle which like MG is biarticular and contributes to both plantar flexion and knee flexion torque (Wolf et al. 1998) but is also known to have a different morphological makeup to MG (Wolf and Kim 1997). The novel aspect of this study was that this modulation can occur under dynamic conditions and discrete recruitment and de‐recruitment thresholds can be identified for different MG MUs based on fascicle length. The observation of a concomitant increase in SOL activation via increases in firing frequency and/or recruitment of additional MUs to overcome the force deficit of a downward regulated and shorter MG while maintaining a constant torque output at the ankle is also novel.

The current experiment takes results well beyond those of earlier studies by associating muscle fascicle length with MG excitability rather than joint position or its associated muscle‐tendon unit length. A recent study by Tokuno et al. (2012) identified that MG fascicle length did not have a direct or one‐to‐one relationship with knee joint angle during passive knee rotations. The MG fascicle behavior presented in that study is very similar to the one shown here, with MG fascicles being at longer lengths during knee flexion compared to knee extension. The dissociation between knee angle (muscle‐tendon unit length) and fascicle length is most likely due to the effect of the relatively compliant Achilles tendon being in series with MG, which is known to exhibit some degree of hysteresis during length cycling (Wren et al. 2003). Changes in short‐range muscle stiffness via activation of contractile tissue may further complicate the relationship between joint angle and muscle fiber length leaving any peripheral control signal difficult for the central nervous system to interpret (Loram et al. 2009; Day et al. 2013).

Why MG MUs de‐recruit at specific fascicle lengths remains a mystery. In this study, MG fascicles shortened on average by approximately 9% over 60^°^ of knee flexion. A decrease of even half that amount will significantly reduce MG's force‐producing capability as a shortening of 10% from its optimal length is known to move MG fascicles onto the lower part of the ascending limb of the active length tension curve (Maganaris 2003; Hoffman et al. 2012). The reduced force‐producing capability of MG is further exacerbated by its negligible amount of passive tension at such shortened lengths (Hoffman et al. 2012). Considering the poor force‐producing capacity of MG at short lengths, it may well be that the central nervous system is attune to muscle fascicle or fiber lengths and regulates the excitability of at least the low threshold MG motoneurones accordingly. Possible mechanisms that could produce such an effect on the MG motoneurone pool excitability would be disfacilitation from shortening MG muscle spindles, reduced MG fusimotor drive or inhibition from other peripheral sources (Golgi tendon organs, joint receptors, etc.). As the observations in this study were made with low levels of plantar flexor torque, we can only speculate on what we would likely observe during higher levels of plantar flexor torque. As fascicle shortening would likely be shorter for higher forces with the same joint configuration, we might expect to see de‐recruitment of MG units occurring earlier during knee flexion and re‐recruited later during knee extension.

An alternative explanation for withdrawal of MG excitation during shortening of its fascicles may be related to unwanted flexion torque being generated about the knee while it is being rotated by the dynamometer, as it may be undesirable to generate knee flexion torques at the same time as ankle plantar flexion moments when the knee is bent. This may be driven by the fact that the moment arm of MG about the knee will increase as the knee becomes more flexed. As there is likely to be a significant relationship between MG fascicle length and MG force development, particularly as passive tension becomes negligible, it may well be that force detecting inputs such as Golgi tendon organs contribute to the decreased excitability of the homonymous MG (MacDonell et al. 2010). To some extent, this helps to explain the overall lower activation of SOL during knee extension as compared to knee flexion, as greater MG force would be produced during knee extension for the same level of MG activation.

The conditions under which the current experiment was conducted are somewhat unique; however, natural movement conditions do exist where the knee moves through similar ranges of motion, leading to the question of whether the observed MG behavior exists during more functional movement‐related tasks. There is some evidence to suggest that during cycling, MG is preferentially activated at longer lengths, while SOL is preferentially recruited at shorter gastrocnemius lengths as MG onset does not occur before the knee moves into extension (Gregor et al. 1991; Neptune et al. 1997). In addition, during sit‐to‐stand movements, MG activity is low compared to SOL until the knee is close to full extension (Doorenbosch et al. 1994). Furthermore, during maximum voluntary plantar flexions performed over a range of different knee angles, Signorile et al. (2002) reported significantly higher levels of MG activation when the knee was fully extended compared to when the knee was flexed at 90°, with SOL showing the opposite behavior. Taken together, the combined results indicate that the triceps surae muscle group is a complex neuromechanical system, whereby the biarticular MG can be activated independently to its monoarticular synergist SOL. The neural mechanism underlying the overall control of the system remains unknown; however, it does appear that there is a strong association of fascicle length to the activation of MG.

## Conclusion

The results of this study show that under dynamic conditions, the drive to MG is modulated such that MU de‐recruitment occurs with progressive MG fascicle shortening and recruitment occurs with progressive lengthening. Furthermore, the results also indicate that muscle length rather than joint angle accounts for the changes in MG recruitment during knee flexion and extension. This could have important consequences for our understanding of the control of multiarticular joint muscles. Whether the same principles of control are utilized in functional movements that might require plantar flexion forces while the knee is bent, such as the sit‐to‐stand movement, or whether similar mechanisms are utilized at other joints, requires further attention.

## Acknowledgments

Benedikt Lauber was supported by the German Academic Exchange Service. The authors thank Dr David Lloyd for his help in producing the model for the experimental setup in Fig. 1.

## Conflict of Interest

None declared.
